# KAI2 regulates seedling development by mediating light‐induced remodelling of auxin transport

**DOI:** 10.1111/nph.18110

**Published:** 2022-04-09

**Authors:** Maxime Hamon‐Josse, José Antonio Villaécija‐Aguilar, Karin Ljung, Ottoline Leyser, Caroline Gutjahr, Tom Bennett

**Affiliations:** ^1^ School of Biology Faculty of Biological Sciences University of Leeds Leeds LS2 9JT UK; ^2^ Plant Genetics TUM School of Life Sciences Technical University of Munich (TUM) Emil Ramann Str. 4 85354 Freising Germany; ^3^ Genetics Faculty of Biology LMU Munich Grosshaderner St. 4 82152 Martinsried Germany; ^4^ Department of Forest Genetics and Plant Physiology Umeå Plant Science Centre Swedish University of Agricultural Sciences SE‐901 83 Umeå Sweden; ^5^ Sainsbury Laboratory Cambridge University Bateman Street Cambridge CB2 1LR UK

**Keywords:** Arabidopsis, auxin, auxin transport, KAI2 signalling, light signalling, PIN proteins, seedling development

## Abstract

- Photomorphogenic remodelling of seedling growth is a key developmental transition in the plant life cycle. The α/β‐hydrolase signalling protein KARRIKIN‐INSENSITIVE2 (KAI2), a close homologue of the strigolactone receptor DWARF14 (D14), is involved in this process, but it is unclear how the effects of KAI2 on development are mediated.
- Here, using a combination of physiological, pharmacological, genetic and imaging approaches in *Arabidopsis thaliana* (Heynh.) we show that *kai2* phenotypes arise because of a failure to downregulate auxin transport from the seedling shoot apex towards the root system, rather than a failure to respond to light *per se*.
- We demonstrate that KAI2 controls the light‐induced remodelling of the PIN‐mediated auxin transport system in seedlings, promoting a reduction in PIN7 abundance in older tissues, and an increase of PIN1/PIN2 abundance in the root meristem. We show that removing PIN3, PIN4 and PIN7 from *kai2* mutants, or pharmacological inhibition of auxin transport and synthesis, is sufficient to suppress most *kai2* seedling phenotypes.
- We conclude that KAI2 regulates seedling morphogenesis by its effects on the auxin transport system. We propose that KAI2 is not required for the light‐mediated changes in PIN gene expression but is required for the appropriate changes in PIN protein abundance within cells.

## Introduction

Once a buried seedling germinates, it first grows heterotrophically on seed reserves and invests energy in reaching the soil–air interface during a developmental phase named skotomorphogenesis. The development pattern during skotomorphogenesis consist of a rapid hypocotyl etiolation, the formation of an apical hook containing the immature cotyledons and protecting the shoot apical meristem, and an inhibition of root growth. Once it reaches the light, the seedling undergoes corresponding photomorphogenic developmental changes, including cessation of hypocotyl elongation, opening of the apical hook, expansion of the cotyledons and development of a competent root system. This is coupled with the production of photosynthetic pigments, and the beginning of light capture and photosynthetic metabolism in the seedling shoot. More generally, photomorphogenesis involves a transition from the ‘cheap’ water‐driven elongation of pre‐existing cells already present in the embryo, to the energy‐expensive generation of new cells and organs by the activity of shoot and root meristems (Sassi *et al*., 2012). The photoreceptors that seedlings use to detect changes in the quantity of different wavelengths of light are well characterised and include PHYTOCHROME and CRYPTOCHROME red and blue light receptors (Franklin & Whitelam, 2004). Less well understood are the mechanisms by which seedlings use this light information across their whole body (including those parts that remain in the dark such as the roots) to produce coherent developmental changes, although a range of recent work has advanced this area considerably.

For instance, it has been shown that sugars arising from newly established photosynthesis serve as essential long‐distance signals promoting root development (Kircher & Schopfer, 2012). Light perception in the cotyledons also triggers translocation of ELONGATED HYPOCOTYL5 (HY5) signalling protein from the shoot to the root through phloem where it acts to regulate root development (Chen *et al*., 2016; Zhang *et al*., 2017). Light perception mechanisms also trigger downstream regulation of hormonal signals that act as further developmental regulators (Symons & Reid, 2003; Gommers & Monte, 2018). A particularly important regulator of seedling skotomorphogenesis and photomorphogenesis is the hormone auxin (indole‐3‐acetic acid, IAA). Auxin status within the seedling is strongly influenced by light signalling, including its biosynthesis, perception and distribution (Halliday *et al*., 2009). In low light, auxin synthesis is positively regulated by PHYTOCHROME INTERACTING FACTOR (PIF) transcription factors (Zhao & Bao, 2021), and drives hypocotyl elongation, at least in part by acidification‐induced changes in cell wall stiffness (Li *et al*., 2021; Lin *et al*., 2021). Auxin is also closely associated with formation and opening of the apical hook (Beziat & Kleine‐Vehn, 2018), and with the phototropic bending of the hypocotyl in either skotomorphogenic or photomorphogenic seedlings (Fankhauser & Christie, 2015). Transfer of seedlings from light to dark induces a strong reduction in auxin delivery to the root system (Sassi *et al*., 2012), suggesting that changes in auxin distribution might also account for photomorphogenic changes in root growth. Overall, because of its all‐pervading influence on plant development, auxin is an excellent candidate to mediate coherent photomorphogenic changes within the seedling.

Arabidopsis *karrikin insensitive2*/*hyposensitive to light* (*kai2*/*htl*) mutants implicate a novel hormonal signal in the control of photomorphogenesis (Sun & Ni, 2011; Waters *et al*., 2012). These mutants have elongated hypocotyls and partly unexpanded cotyledons when grown in the light, indicating a deficiency in photomorphogenesis. KAI2 is an α/β‐fold hydrolase receptor protein that is ubiquitous in land plants (Waters *et al*., 2012; Bythell‐Douglas *et al*., 2017), which was identified as mediating perception of smoke‐derived ‘karrikin’ molecules in flowering plants (Waters *et al*., 2012). However, this is not likely to be the ancestral function of KAI2 (Waters *et al*., 2015), leading to the hypothesis that KAI2 is a receptor for an as‐yet‐unidentified endogenous ‘KAI2‐ligand’ (KL) that, despite considerable efforts, remains enigmatic (Conn & Nelson, 2016; Sun *et al*., 2016). Strengthening this idea, KAI2 is closely related to the α/β‐fold hydrolase protein DWARF14, which acts as the receptor for endogenous strigolactone molecules. KAI2 can bind to, and be activated by non‐naturally occurring stereoisomers of strigolactones, but not by naturally occurring stereoisomers (Scaffidi *et al*., 2014). Irrespective of its ligand, KAI2 has an unambiguous downstream signalling pathway in all plants (Machin *et al*., 2020). This pathway is very similar to, and indeed overlapping with, the strigolactone signalling pathway, although the KAI2 signalling pathway appears to be more ancient, with strigolactone signalling only arising in seed plants (Bythell‐Douglas *et al*., 2017; Walker *et al*., 2019). Both D14 and KAI2 act through the SCF^MAX2^ ubiquitin ligase complex to recruit and induce ubiquitination and proteolysis of SUPPRESSOR OF MAX2 1‐LIKE (SMXL) proteins (reviewed in Machin *et al*., 2020). D14 primarily promotes degradation of SMXL6, SMXL7 and SMXL8, although SMXL2 can also be targeted (Soundappan *et al*., 2015; Liang *et al*., 2016; Wang *et al*., 2020b), while KAI2 primarily promotes degradation of SMAX1 and SMXL2 (Khosla *et al*., 2020; Wang *et al*., 2020b). SMXL proteins are still somewhat enigmatic in function; although they certainly seem to influence transcriptional responses, they are not transcription factors themselves, and they may influence different responses through different adapter proteins (Soundappan *et al*., 2015; Wang *et al*., 2015; Song *et al*., 2017; Machin *et al*., 2020; Wang *et al*., 2020a).

Despite considerable interest in its function, the role of KAI2 signalling in seedling photomorphogenesis remains poorly understood. KAI2 signalling is certainly not needed for light perception *per se*, but is required for a normal photomorphogenetic development of the seedlings (Sun & Ni, 2011; Water *et al*., 2012; Lee *et al*., 2019). While cross‐regulation between KAI2 and a subset of light‐responsive genes such as HY5 has previously been suggested to account for these effects (Sun & Ni, 2011) the evidence is not conclusive (Waters & Smith, 2013). We recently showed that KAI2 signalling also acts as a central regulator of seedling root growth, and that an impairment in KAI2 signalling leads to increased lateral root development and retarded root hair formation in young seedlings of Arabidopsis (Villaécija‐Aguilar *et al*., 2019), suggesting that KAI2 signalling may mediate coherent photomorphogenic developmental effects across the seedling. Given the central role of auxin in seedling photomorphogenesis, and the ability of the closely related D14‐mediated strigolactone signalling pathway to regulate auxin distribution by remodelling auxin transport in the shoot (Crawford *et al*., 2010; Shinohara *et al*., 2013; Soundappan *et al*., 2015; Bennett *et al*., 2016b), we hypothesised that KAI2 signalling regulates seedling development by modulating auxin transport in response to light cues.

## Materials and Methods

### Plant materials

*Arabidopsis thaliana* (L.) Heynh. genotypes were in Columbia‐0 (Col‐0) or Landsberg *erecta* (L*er*) parental backgrounds. The *max2‐1* (Stirnberg *et al*., 2002), *max2‐8* (Nelson *et al*., 2011), *max4‐5* (Bennett *et al*., 2006), *d14‐1* (Waters *et al*., 2012), *kai2‐1* (null allele of KAI2, carrying *a* G133E missense mutation) (Waters *et al*., 2012), *kai2‐2* (knock‐out allele of KAI2, carrying a *Ds* insertion in the first intron) (backcrossed 6× to Col‐0) (Bennett *et al*., 2016a), *smax1‐2 smxl2‐1* (Stanga *et al*., 2016), *smxl6‐4 smxl7‐3 smxl8‐1* (Soundappan *et al*., 2015), *DR5v2:GFP* (Liao *et al*., 2015), *pin3‐3 pin4‐3 pin7‐1* (Bennett *et al*., 2016b), *PIN1:PIN1‐GFP* (Benkova *et al*., 2003), *PIN3:PIN3‐GFP*, *PIN4:PIN4‐GFP*, *PIN7:PIN7‐GFP* (Blilou *et al*., 2005), *PIN2:PIN2‐GFP* (Xu & Scheres, 2005), lines have all been previously described.

New genotypes were assembled by crossing relevant existing genotypes and the required homozygous lines were identified using visible, fluorescent or selectable markers or using PCR genotyping. The *kai2‐1 PIN1:PIN1‐GFP* and *kai2‐1 PIN2:PIN2‐GFP* lines were constructed using a *kai2‐1* allele backcrossed four times into Col‐0, rather than the original *kai2‐1* allele in L*er*.

### Growth conditions

Seedlings for phenotypic analysis, dissections, pharmacological treatments, auxin quantification, qPCR and confocal imaging were grown in axenic culture. Seeds were surface sterilised using a 2 h vapour sterilisation method (3 ml of HCl 37% in 100 ml bleach), then sown onto 0.8% agar‐solidified *Arabidopsis thaliana* salts (ATS) medium (pH 5.6) (Wilson *et al*., 1990) with 1% (w/v) sucrose, in square Petri dishes (12 × 12 cm, 60 ml media per plate), and stratified in the dark at 4°C for 2–3 d.

For plants grown in normal light conditions, plates were oriented vertically, and seedlings grown for 6–10 d in a growth chamber under a 16 h : 8 h, light : dark cycle (20°C : 18°C) with light provided by fluorescence tubes (120 μmol m^−2^ s^−1^), both root and shoot tissues were equally exposed to the light. In experiments with transfer from dark‐to‐light conditions, after stratification, plates were placed for 8 h at 120 μmol m^−2^ s^−1^ light/20°C to promote germination and then placed in complete darkness for 4 d at 20°C in a black plastic box in a growth chamber. After 4 d in darkness plates were transferred in the normal light conditions described previously (16 h : 8 h, light : dark cycle (20°C : 18°C)/120 μmol m^−2^ s^−1^), for an additional 1–6 d of growth and both root and shoot tissues were equally exposed to the light.

### Phenotypic analysis

Measurements of seedlings were made at various time points described in the text. A dissecting microscope was used to count emerged adventitious and lateral roots in each root system. Plants were then imaged using a flatbed scanner, and primary root and hypocotyl length was quantified from the resulting images using Fiji (https://imagej.net/Fiji/Downloads). Lateral root density was quantified as the number of lateral roots per mm of primary root. Lateral root and adventitious root primordia numbers were scored by observing DR5v2:GFP as a primordia marker in Col‐0 and *kai2*‐2 with a laser‐scanning confocal microscope LSM880 upright (please refer to the laser microscopy section).

For analysis of root hair length, *Arabidopsis thaliana* seeds were grown in axenic conditions on 12 × 12 cm square Petri dishes containing 60 ml in half‐strength Murashige and Skoog medium, pH 5.8 (Duchefa, Haarlem, the Netherlands), supplemented with 1% sucrose and solidified with 1.5% agar. before sawing seeds were surface sterilised by washing with 1 ml of 70% (v/v) ethanol and 0.05% (v/v) Triton X‐100 with gentle mixing by inversion for 6 min at room temperature, followed by one wash with 96% ethanol and three washes with sterile distilled water. Plants were stratified at 4°C for 3 d in the dark, and then transferred to a growth cabinet at 22°C and placed vertically under light provided by fluorescence tubes (120 µmol m^−2^ s^−1^) for 3 h to promote germination. Subsequently they were grown in the same growth chamber for 5 d in complete darkness in a black plastic box, in light (16 h : 8 h, light : dark cycle), or in dark : light (16 h : 8 h, light : dark cycle) with roots covered with a black paper to keep them in dark, while the aerial part remained illuminated.

For the root hair measurements, root images were taken at 5 d postgermination with a Zeiss Stereo Discovery V8 microscope (Carl Zeiss, Jena, Germany) equipped with a Zeiss Axiocam 503 colour camera (Zeiss). The number of root hairs was determined by counting the root hairs between 2 and 3 mm from the root tip on each root, and root hair length was measured for 10 root hairs per root in a minimum of eight roots per genotype and condition using Fiji (https://imagej.net/Fiji/Downloads) according to Villaécija‐Aguilar *et al*. (2021b).

For the root apical meristem size, root were mounted on glass slides and stained with propidium iodide (PI). PI excitation was performed using a 561 nm laser, and fluorescence was detected between above 610 nm. RAM size was measured using Fiji straight segment tool as the length from the first nondividing cortical cell near the elongation zone and the cells forming the quiescent centre.

### Seedling dissections

Here, 4‐d‐old etiolated seedlings were dissected *in situ* on agar plates using a very sharp scalpel. Decapitation assays were performed by removing the seedling meristem and cotyledons at the junction of the cotyledons and hypocotyl.

### Pharmacological experiments

For pharmacological experiments, 1000× stock solutions 1*‐N‐*naphthylphthalamic acid (NPA) (Duchefa) and l‐kynurenine (Sigma‐Aldrich) were made by dissolving the appropriate mass of the compound in a 2% DMSO and 70% ethanol solution. From these stocks, a 60 µl aliquot per plate was added to hand‐warm ATS‐agar medium before pouring the plates. Control plates contained 60 µl per plate of 2% DMSO and 70% ethanol solvent control solution. Seed were either germinated directly on plates containing to the pharmacological treatments, or were transferred after initial growth on plain plates, as indicated in the text.

### Free IAA determination

For the first experiment (Fig. 2f), seedlings were grown for 4 d on ATS‐agar medium with sucrose in the dark, and then transferred to the light. Some seedlings were dissected immediately, with the cotyledons separated from the hypocotyl + roots, and flash frozen in liquid nitrogen. The other seedlings were dissected after a further 1 d of growth in the light, and then dissected in the same way. There were four biological samples for each genotype and time point, each containing pooled tissue from 60 seedlings. For the second experiment (Fig. 2g) seedlings were grown for 6 d on ATS‐agar medium with sucrose in the light, at which point the roots were dissected from the seedlings, and flash frozen. There were four biological samples for each genotype and time point, each containing pooled tissue from 60 seedlings. From these samples (10–20 mg fresh weight) IAA was purified and analysed by gas chromatography‐tandem mass spectrometry (GC‐MS/MS) as described in Andersen *et al*. (2008) with minor modifications. To each sample, 500 pg ^13^C_6_‐IAA was added as an internal standard before extraction.

### Auxin transport assay

For the auxin transport assay, *Arabidopsis thaliana* seed sterilisation and seedling growth were performed as described for root hair measurements in light conditions. An agar droplet containing 100 nM ^3^H‐IAA (Hartmann analytic) and solvent (DMSO) or 10 µM NPA (Olchemim) in DMSO was applied below the aligned root–shoot junctions of 5 d postgermination Arabidopsis seedlings. At 18 h after the treatment, the amount of radioactivity was quantified in a 5‐mm apical segment as previously described in Lewis & Muday (2009).

### *Laser*‐*scanning confocal microscopy*

To visualise fluorescent reporter lines, laser‐scanning confocal microscopy was performed on either a Zeiss LSM700 or a LSM880 imaging system with a ×20 lens. Tissues were stained with PI (10 µg ml^−1^) and mounted on glass slides. Green fluorescent protein (GFP) excitation was performed using a 488 nm laser, and fluorescence was detected between 488 and 555 nm. PI excitation was performed using a 561 nm laser, and fluorescence was detected between above 610 nm. The same detection settings were used for all images captured in a single experiment. GFP quantification was performed on nonsaturated images, using Zeiss Zen software.

For the different GFP lines (DR5v2:GFP, PIN1‐GFP, PIN2‐GFP, PIN3‐GFP, PIN4‐GFP and PIN7‐GFP) fluorescence was quantified in regions of interest (Supporting Information Fig. S3e,f) either in the hypocotyl, the shoot–root junction, the older differentiated zone (ODZ; between the first two emerged lateral roots (LR)), the middle differentiated zone (MDZ) between the last emerged LR and the first LR primordia), the young differentiated zone (YDZ, in the root hair elongation zone), or in the meristem zone (MZ) including columella and quiescent centre nuclei as appropriate. For *DR5v2:GFP*, the fluorescence intensity is plotted as the mean GFP intensity measured in 5–10 nuclei/seedling in the region of interest. For PIN1, fluorescence intensity was plotted as the mean GFP intensity measured in 5–10 basal plasma membranes/seedling in the region of interest (stele cells above the RAM). For PIN2, fluorescence intensity was plotted as the mean GFP intensity measured in 5–10 apical plasma membranes (PM)/seedling in the epidermal cells of the MZ. For PIN1‐GFP and PIN2‐GFP, the PM were selected using the ImageJ segmented line tool with a ‘line width of 5’. For PIN3‐GFP and PIN7‐GFP, fluorescence intensity was plotted as the mean GFP intensity measured in a rectangle of 40 × 80 µm (width × height) covering the region of interest. For PIN3‐GFP and PIN7‐GFP in the MZ, fluorescence intensity was plotted as the mean GFP intensity measured at the plasma membrane of columella cells (selected using the ImageJ segmented line tool with a ‘line width of 5’).

### RNA extraction and gene expression analysis

For expression analysis of PIN genes, Col‐0 and *kai2‐2* seedlings were grown for 4 d on ATS‐agar medium with sucrose in the dark, and then transferred to the light for 0, 1 or 3 d of additional growth. For each time point and genotype, three biological samples were collected by pooling *c*. 16 seedlings, which were then flash frozen in liquid nitrogen. Total RNA was extracted using RNeasy Plant Mini kits (Qiagen), and then DNase treated using the Turbo DNA‐free kit (Ambion), both as per manufacturer’s instructions. RNA was quantified using a NanoDrop 1000 spectrophotometer. For cDNA synthesis, Superscript (Invitrogen) II was used to reverse transcribe 500 ng of total RNA according to the manufacturer’s instructions. Quantification of transcript levels was carried out using SYBR Green reactions with 5 ng cDNA in a 20 μl volume on a Light Cycler 480 II (Roche) relative to the reference gene *UBC10* (*POLYUBIQUITIN10*, At4g05320). Three technical replicates were run for each biological replicate and averaged. Calculation of the expression levels was done using the ΔΔCt method (Czechowski *et al*., 2005). Primers used were:

*PIN1*‐F: 5′‐CAGTCTTGGGTTGTTCATGGC‐3′; *PIN1‐*R*:* 5′‐ATCTCATAGCCGCCGCAAAA‐3′

*PIN3*‐F: 5′‐CCATGGCGGTTAGGTTCCTT‐3′; *PIN3*‐R: 5′‐ATGCGGCCTGAACTATAGCG‐3′

*PIN4*‐F: 5′‐AATGCTAGAGGTGGTGGTGATG‐3′; *PIN4*‐R: 5′‐TAGCTCCGCCGTGGAATTAG‐3′

*PIN7*‐F: 5′‐GGTGAAAACAAAGCTGGTCCG‐3′; *PIN7‐*R: 5′‐CCGAAGCTTGTGTAGTCCGT‐3′

UBQ10‐F: 5′‐GGTTTGTGTTTTGGGGCCTTG‐3′; UBQ10‐R: 5′‐CGAAGCGATGATAAAGAAGAAGTTCG‐3′

### Statistical analyses

Data generally show independent biological replicates, although in a small number of cases we pooled data from two replicates, when the distributions of data between the replicates were sufficiently similar. Statistical analyses were performed in Rstudio and GraphPad Prism (9.3.1). If comparing two groups, we used the *t*‐test with Welch’s correction (unequal variances *t*‐test) with *P*‐values (*, *P* ≤ 0.05; **, *P* ≤ 0.01; ***, *P* ≤ 0.001) indicating differences between genotype or conditions, as appropriate. If the comparison contained more than two groups, we used one‐way analysis of variance (ANOVA), followed by Tukey’s honest significant difference (HSD) *post hoc* test (95% confidence interval (CI)), with groups showing statistical differences indicated by different letters. The test(s) performed for each graph are indicated in the legend.

## Results

### KAI2 mediates light‐induced remodelling of seedling development

As *kai2* mutants display stronger phenotypes in younger seedlings, particularly in the roots (Villaécija‐Aguilar *et al*., 2019), we hypothesised that *kai2* phenotypes arise from sluggish adaptation to the light, rather than a long‐term inability to grow correctly in the light. All seedlings undergo a dark‐to‐light, photomorphogenic transition after germination, although this can be delayed by germinating plants in the dark, triggering a prolonged skotomorphogenic state with an etiolated hypocotyl. To test the role of KAI2 in light adaptation, we used both seedlings grown under standard light conditions (immediate photomorphogenesis, indicated by (L) in the corner of a figure panel) and seedlings pretreated by growth in the dark to promote hypocotyl etiolation (delayed photomorphogenesis, indicated by (D : L) in the corner of a figure panel). This latter etiolation/de‐etiolation system has advantages in terms of synchronising experimental time points and imaging of hypocotyls, but both set‐ups allowed us to study the role of KAI2 in photomorphogenic, dark‐to‐light transition because *kai2* seedlings showed obvious growth defects under both sets of conditions.

**Fig. 1:**
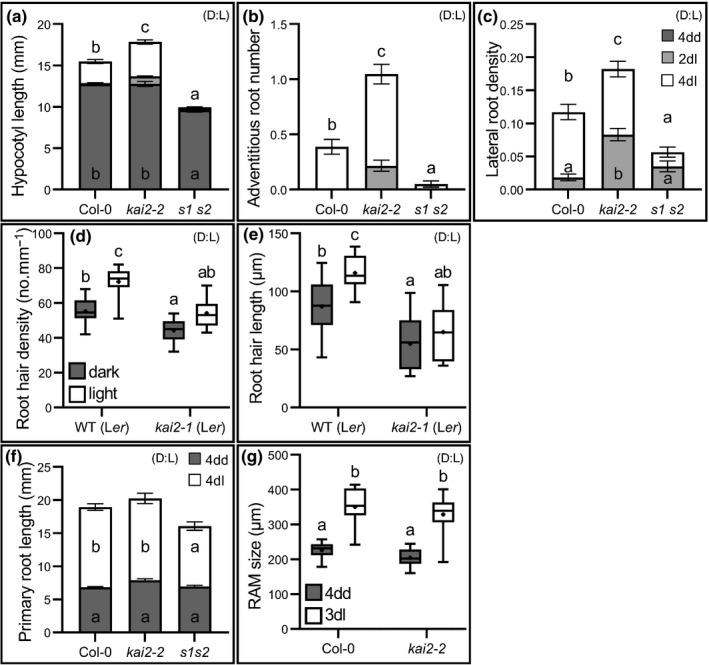
KAI2 mediates light‐induced remodelling of seedling development. For all figure panels (D) indicates that *Arabidopsis thaliana* plants were grown in continuous darkness, (L) indicates that plants were grown under a standard light regime (16 h : 8 h, light : dark), and (D : L) indicates that plants were grown in continuous darkness for some days before transfer to standard light conditions. (a–c) Hypocotyl length (a), adventitious root number (b), and lateral root density (c) in wild‐type (Col‐0), *kai2‐2* and *smax1‐2 smxl2‐1* (*s1 s2*) seedlings at 4 d growth in the dark (4dd), and after subsequent transfer to normal light conditions for 2 and 4 d (2dl and 4dl). Data are from two independent replicates pooled together (*n* = 43–91 seedlings per genotype and condition); a third independent replicate gave comparable results. Error bars represent ± SE. (d, e) Root hair density (c) and length (d) in 5‐d‐old seedlings grown in the dark (dark), or in normal light conditions (light). Data correspond to one experimental replicate (*n* = 8–10 seedlings per genotype and condition); a second independent experimental replicate gave comparable results. (f, g) Primary root length (f), and root apical meristem size (RAM) (g) in wild‐type (WT) (Col‐0), *kai2‐2*, and *smax1‐2 smxl2‐1* (*s1 s2*) seedlings at 4 d growth in the dark (4dd), and after subsequent transfer to normal light conditions for 3 or 4 d (3dl and 4dl). (f) Data correspond to two independent experimental replicates pooled together (*n* = 43–91 seedlings per genotype and condition); a third independent experimental replicate gave comparable results. Error bars represent ± SE. (g) One experiment was performed (*n* = 8–10 seedlings per genotype and time point). (d–g) The boxes in the box plot show the lower and upper quartiles and median values, mean is represented as (•), whiskers show minimal and maximal data values. (a–g) Statistical groups indicated by letters were determined by one‐way ANOVA with *post hoc* Tukey’s honest significant difference (HSD) (95% confidence interval (CI)), different letters indicate statistical differences between groups.

To determine the precise roles of KAI2 in growth adaptation to light, we grew wild‐type (Col‐0) and *kai2‐2* seedlings in the dark for 4 d (4dd), before tracking their development for 2 d (4dd/2dl) and 4 d (4dd/4dl) after the transition to the light. In wild‐type seedlings, the transfer to the light causes a rapid cessation of hypocotyl growth by 4dd/2dl, followed by a slight increase in hypocotyl growth by 4dd/4dl (Fig. 1a). Consistent with previous studies, we found that *kai2* mutants have a wild‐type hypocotyl phenotype in the dark but failed to stop growing after transition to light between 4dd and 4dd/2dl (Fig. 1a). We observed that neither wild‐type nor *kai2* seedlings initiated adventitious or lateral roots by 4dd, and only started initiating roots after transfer to the light (Fig. 1b,c). We observed that *kai2* mutants produced more adventitious roots than wild‐type between 4dd and 4dd/2dl (Fig. 1b), at the level of both initiation and emergence (Fig. S1d). We also observed that lateral root formation was greater in *kai2* than wild‐type between 4dd and 4dd/2dl, but sustained outgrowth does not differ significantly (Fig. 1c). This phenotype arises because *kai2* mutants initiated more lateral root primordia, which also showed increased emergence in comparison with wild‐type (Fig. S1e). In terms of root formation, the *kai2* mutants therefore do not under‐respond to light as they do in the hypocotyl. Rather, they actually initially over‐respond to light exposure by initiating too many lateral/adventitious roots, before adopting the same growth pattern as wild‐type over the longer term. Growth in light conditions also promoted an increase in root hair (RH) density and RH length in wild‐type compared with dark‐grown seedlings (Fig. 1d,e). By contrast, *kai2* mutants have shorter root hair and reduced density in the dark, and the mutants lack the ability to correctly adjust the RH development in response to the light status of the root system (Fig. 1d,e).

It is notable that the increased adventitious roots seen in *max2* mutants, which lack both KAI2 and strigolactone signalling (Waters *et al*., 2012), were previously reported to be the result of decreased strigolactone signalling (Rasmussen *et al*., 2012). A more recent report has suggested that KAI2 signalling is more important for this phenotype than strigolactone signalling (Swarbreck *et al*., 2020), and our results are consistent with this; we also observed increased adventitious roots in light‐grown *kai2* seedlings relative to wild‐type, rather than in strigolactone mutants (Fig. S1a). This was consistent across *kai2* mutants in the Col‐0 and Ler backgrounds (Fig. S1b).

We also grew the *smax1‐2 smxl2‐1* (*s1s2*) mutant lacking the proteolytic targets of KAI2 activity (Khosla *et al*., 2020). The *s1s2* mutant phenotypes are similarly complex; *s1s2* hypocotyls seem to over‐respond to light exposure, but they do not initiate adventitious roots in the light, while the number of lateral roots initially increased strongly, but failed to show a sustained increase (Fig. 1a–c). Correspondingly the *max2* adventitious root phenotype was suppressed by mutations in *SMAX1* and *SMXL2* (Fig. S1c). Taking the phenotypes of *kai2* and *s1s2* mutants together, the overall role of KAI2 signalling in photomorphogenesis therefore seems to be more concerned with the correct spatial patterning of growth responses, rather than the response to light *per se*.

### KAI2 modulates auxin distribution in the seedling

**Fig. 2:**
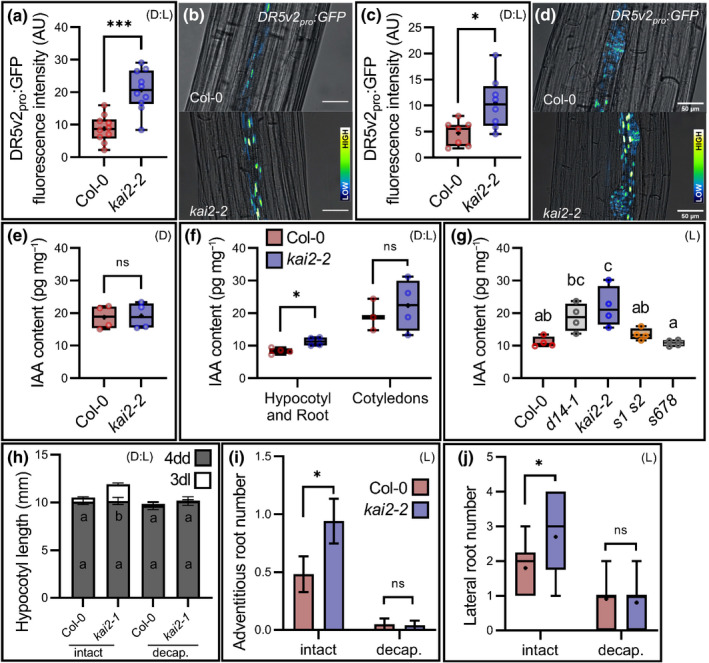
KAI2 modulates auxin distribution in the seedling. For all figure panels (D) indicates that *Arabidopsis thaliana* plants were grown in continuous darkness, (L) indicates that plants were grown under a standard light regime (16 h : 8 h, light : dark), and (D : L) indicates that plants were grown in continuous darkness for some days before transfer to standard light conditions. (a–d) Auxin response (average *DR5v2:GFP* fluorescence intensity) in Col‐0 and *kai2* seedlings grown 4 d in the dark followed by 4 d of normal light‐growth, in the hypocotyl (a, b) and adventitious root primordia (c, d). (a, c) Green fluorescent protein (GFP) quantification in the hypocotyl and in the adventitious root primordia, respectively. For (a) data correspond to the average GFP intensity of 10 seedlings per genotype, in which each sample was the average of 10 nuclei per hypocotyl, from one experimental replicate; a second independent experimental replicate gave comparable results. (c) Data correspond to the average GFP intensity of seven or eight adventitious root primordia of similar stage (from four or five seedlings per genotype), and each sample was the average of 10 nuclei, from one experimental replicate; a second independent experimental replicate gave comparable results. *, *P* ≤ 0.05; ***, *P *≤ 0.001; indicate differences compared with wild‐type (Welch’s *t*‐test). (b, d) Representative microscopy images with bright field (grey) and GFP signals represented with false colour, with dark blue as low signal intensity and bright white as high signal intensity. Bar, 50 µm. (e–g) Indole‐3‐acetic acid (IAA) quantification (pg IAA per mg of tissue, pg mg^−1^) in whole seedlings grown for 4 d in the dark (e), or roots grown 5 d under normal light conditions (g), or in cotyledons and hypocotyl/root sections of seedlings grown for 4 d in the dark and subsequently transferred to normal light conditions for 1 d (f). (*n* = 3–4 pools of 30 seedlings). Statistical groups indicated by letters were determined by one‐way ANOVA with *post hoc* Tukey’s honest significant difference (HSD) (95% confidence interval (CI)). *, *P* ≤ 0.05; ***, *P* ≤ 0.001; indicate differences compared with wild‐type (Welch’s *t*‐test). ns, no significant difference. (h) Measurement of hypocotyl length in Col‐0 and *kai2‐1* seedlings grown for 4 d in the dark (4dd) before subsequently undergoing apex decapitation or left intact before transfer to normal light conditions for 3 d (3dl). Stacked bars indicate length before treatment after 4dd (grey) and additional growth in the light after treatment (3dl, white). Data correspond to one experimental replicate (*n* = 12–14 seedlings per genotype); a second independent experimental replicate gave comparable results. Statistical groups indicated by letters were determined by one‐way ANOVA with *post hoc* Tukey HSD (95% confidence interval (CI)). (h, i) Error bars represent ± SE. (i, j) Measurement adventitious root number (i), and lateral root number (j) of seedlings grown for 4 d in the dark and subsequently undergoing apex decapitation or left intact before transfer to normal light conditions for 3 d. Data correspond to one experimental replicate (for (i) *n* = 20–32 seedlings per genotype, for (j) *n* = 10–11 seedlings per genotype), a second independent experimental replicate gave comparable results. *, *P* ≤ 0.05; ***, *P* ≤ 0.001; indicates differences compared with wild‐type (Welch’s *t*‐test). ns, no significant difference. (a, c, e–g) The boxes in the box plot show the lower and upper quartiles and median values (horizontal line), mean is represented as (•), whiskers show minimal and maximal data values.

Given the prominent role of auxin in hypocotyl elongation and root growth (Jensen *et al*., 1998; Lavenus *et al*., 2013), and its known roles downstream of light perception (Casal *et al*., 2013; Fankhauser & Christie, 2015), we hypothesised that the *kai2* phenotype might arise due to perturbations in auxin response. To test this idea, we examined the expression of the *DR5v2:GFP* auxin reporter in the relevant tissues. We found that the auxin response was increased in the hypocotyls, adventitious root primordia and proliferating lateral roots in *kai2*, consistent with the idea that the auxin response was perturbed in these mutants (Figs 2a–d, S1, S2a,b). We tested whether this altered response might be caused by increased auxin abundance in *kai2* mutants, by directly measuring auxin levels. In seedlings dark grown for 4 d, auxin levels in *kai2* and wild‐type were identical (Fig. 2e) but, in 4dd/1dl seedlings, auxin levels in *kai2* seedlings were reproducibly higher than in the wild‐type. These data could therefore be consistent with increased auxin abundance causing the *kai2* phenotype. Interestingly, however, this increase was larger in the hypocotyl/root compartment compared with the cotyledons (Fig. 2f). It is also notable that in the roots of 5‐d‐old light‐grown (5dl) seedlings, auxin levels were similarly increased in *kai2* mutants relative to wild‐type, but so were levels in the *d14‐1* SL receptor mutant, which does not have the same root phenotypes as *kai2‐2* (Fig. 2g) (Villaécija‐Aguilar *et al*., 2019). Conversely, the *s1s2 (smax1‐2 smxl2‐1)* and *smxl6‐4 smxl7‐3 smxl8‐1* (*s678*) triple mutant (which lacks the proteolytic targets of D14 activity) had the same auxin levels as the wild‐type, but had dramatic root phenotypes not present in the wild‐type (Villaécija‐Aguilar *et al*., 2019). Therefore, changes in auxin abundance alone were unable to explain the specific phenotypes observed in the *kai2* and *smax1 smxl2* mutants.

Given the altered partitioning of auxin abundance in *kai2* mutants at 4dd/1dl (Fig. 2f), we reasoned that the effects of KAI2 on seedling development might relate more to altered auxin distribution than to simply increased auxin levels. Our data (Fig. 2a–g) showed a higher auxin abundance and signalling in the upper part of the *kai2* mutant seedlings (in the hypocotyl and upper part of the root), suggesting that this is the direct cause for higher growth rate in these regions. Next, we hypothesised that, given the well known rootward transport of auxin in plant tissues, the shoot apex could be the source for this local auxin accumulation (Fig. 1). To test this idea, we first used microsurgical approaches. We decapitated wild‐type and *kai2‐2* seedlings at 4dd by removing the seedling apex, and then assessed their phenotype at 4dd/3dl. This treatment was sufficient to restore the *kai2* hypocotyl, adventitious root, and lateral root phenotypes to wild‐type (Figs 2h–j, S2d,e), consistent with apically‐derived auxin driving these effects. Therefore, the phenotype of *kai2* appearsed to be associated with increased rootward auxin flux, similar to the changes in auxin transport observed in strigolactone mutants (Bennett *et al*., 2006, 2016a; Shinohara *et al*., 2013). Notably, the increased rootward auxin transport in strigolactone mutants did not deplete the shoot of auxin, presumably because the increased export of auxin was balanced by increased auxin synthesis in source tissues (Bennett *et al*., 2006; Prusinkiewicz *et al*., 2009). The same phenomenon seems to apply in kai2 seedlings, as the increase in auxin in the root did not result in a reduction in auxin in the shoot apex (Fig. 2f).

### Auxin transport in the seedlings is remodelled at the dark–light transition

**Fig. 3:**
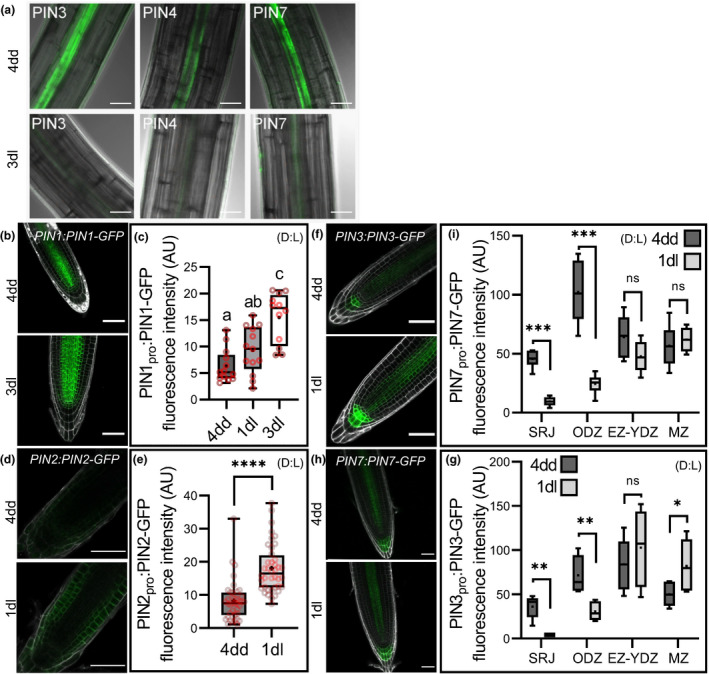
Remodelling of auxin transport at the dark–light transition. For all figure panels (D) indicates that *Arabidopsis thaliana* plants were grown in continuous darkness, (L) indicates that plants were grown under a standard light regime (16 h : 8 h, light : dark), and (D : L) indicates that plants were grown in continuous darkness for some days before transfer to standard light conditions. (a) PIN3‐GFP, PIN4‐GFP, and PIN7‐GFP abundance at the very basal end of hypocotyls of wild‐type seedlings after 4 d growth in the dark (4dd, top row), and subsequent transfer to normal light conditions for 3 d (3dl, bottom row). Images overlay bright field (grey) and green fluorescent protein (GFP) signals (green). Bar, 30 µm. (b, d, f, h) PIN1‐GFP, PIN2‐GFP, PIN3‐GFP, and PIN7‐GFP abundance in meristem zone (MZ) of wild‐type seedlings after 4 d growth in the dark (4dd, top row), and subsequent transfer to normal light conditions for 1 or 3 d (1dl, 3dl). Microscopy images overlay propidium iodide (grey) and GFP signals (Green). Bar, 50 µm. (c, e) Quantification of PIN1pro:PIN1‐GFP (c) and PIN2pro:PIN2‐GFP (e) signals in meristem zone (MZ) of wild‐type seedlings after 4 d growth in the dark (4dd), and subsequent transfer to normal light conditions for 1 or 3 d (1dl, 3dl). For (c) data correspond to the averaged PIN1‐GFP intensity in the MZ from one experimental replicate (*n* = 12–13 seedlings per genotype and time point); two other independent experimental replicates gave comparable results. For (e) data correspond to the averaged PIN2‐GFP intensity in the apical plasma membrane of MZ epidermal cells from one experimental replicate (*n* = 39–40 plasma membranes from four seedlings for each genotype and time point). Statistical groups indicated by letters were determined by one‐way ANOVA with *post hoc* Tukey HSD (95% confidence interval (CI)). *, *P* ≤ 0.05; **, *P* ≤ 0.01; ***, *P* ≤ 0.001; ****, *P* ≤ 0.0001; indicates differences compared with wild‐type (Welch’s *t*‐test). (g, i) Quantification of PIN3‐GFP (g) and PIN7‐GFP (i) signals in the shoot–root junction (SRJ), older differentiation zone (ODZ), junction of the elongation and young differentiation zone (EZ‐YDZ), and meristem zone (MZ) of wild‐type seedlings after 4 d growth in the dark (4dd), and subsequent transfer to normal light conditions for 1 d (1dl). Data correspond to one experimental replicate (*n* = 4–7 seedlings per genotype and time point); for (g) a second independent experimental replicate gave comparable results. *, *P* ≤ 0.05; **, *P* ≤ 0.01; ***, *P* ≤ 0.001; indicates differences compared with wild‐type (Welch’s *t*‐test). ns, no significant difference. (c, g, e, i) The boxes in the box plot show the lower and upper quartiles and median values, mean is represented as (•), whiskers show minimal and maximal datavalues.

**Fig. 4:**
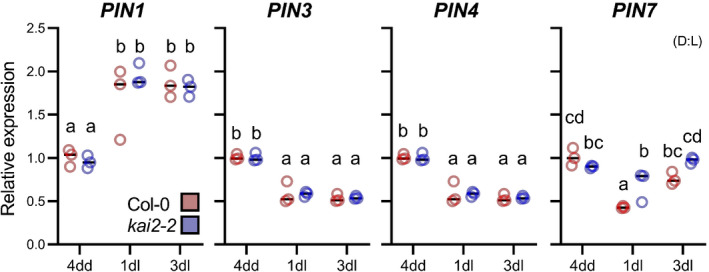
Transcriptional remodelling of auxin transport at the dark–light transition. Expression of *PIN* genes in *Arabidopsis thaliana* relative to the reference gene *UBC10*, in wild‐type and *kai2‐2* seedlings after 4 d growth in the dark (4dd), and subsequent transfer to normal light conditions for 1 and 3 d (1dl, 3dl). For each gene, expression is normalised to the expression in wild‐type at 4dd. *n* = 3 biological samples collected by pooling *c*. 16 seedlings per genotype and time point. Statistical groups indicated by letters were determined by one‐way ANOVA with *post hoc* Tukey’s honest significant difference (HSD) (95% confidence interval (CI)). Black lines represent mean.

Given these results, we hypothesised that the altered auxin transport in *kai2* might be caused by increased abundance of members of the PIN family of auxin efflux carriers, which play major roles in mediating directional auxin transport (Adamowski & Friml, 2015), and have been implicated in the phenotypic effects of strigolactone signalling (Bennett *et al*., 2006, 2016a; Shinohara *et al*., 2013; Zhang *et al*., 2020). We examined the abundance of PIN proteins in wild‐type hypocotyls in the dark (4dd) and after transition to the light (4dd 3dl) using GFP protein fusions. PIN3, PIN4 and PIN7 are all highly abundant at 4dd, but greatly reduced by 4dd/3dl (Fig. 3a). We also examined PIN protein abundance in roots. Consistent with previous reports, we found that PIN2 accumulation was induced by transfer to light (Laxmi *et al*., 2008) (Fig. 3d,e). We found that PIN1 abundance also increased in the root meristem after exposure to light (Fig. 3b,c). PIN4 was difficult to detect, but PIN3 and PIN7 are highly abundant throughout the length of 4dd roots (Fig. 3f,h). In older root tissues, their abundance declined by 4dd/1dl but, in the root meristem and elongation zone, their abundance was maintained or increased (Fig. 3g,i). Therefore, after the dark–light transition, there is a major re‐organisation of the seedling auxin transport network. Expression of the PIN genes, as assessed by qRT‐PCR on whole seedlings, reflected these changes in PIN abundance, with *PIN3*, *PIN4* and *PIN7* all downregulated at 4dd/1dl and 4dd/3dl relative to 4dd (Fig. 4). Conversely, PIN1 was upregulated at 4dd/1dl and 4dd/3dl relative to 4dd (Fig. 4), consistent with the observations of Sassi *et al*. (2012).

We questioned whether these changes in PIN protein expression at the dark–light transition also resulted in observable changes in auxin distribution and/or response, as visualised by the *DR5v2:GFP* reporter. In hypocotyls, we observed a dramatic downregulation of *DR5* signal between dark‐grown (4dd) seedlings and those transferred to the light (4dd1dl) (Fig. S3a,b), while in the root apical meristem we observed a gradual increase of DR5 signal between 4dd and 4dd1dl and 4dd3dl seedlings (Fig. S3c,d).

### KAI2 regulates light‐induced remodelling of PIN‐mediated auxin transport

**Fig. 5:**
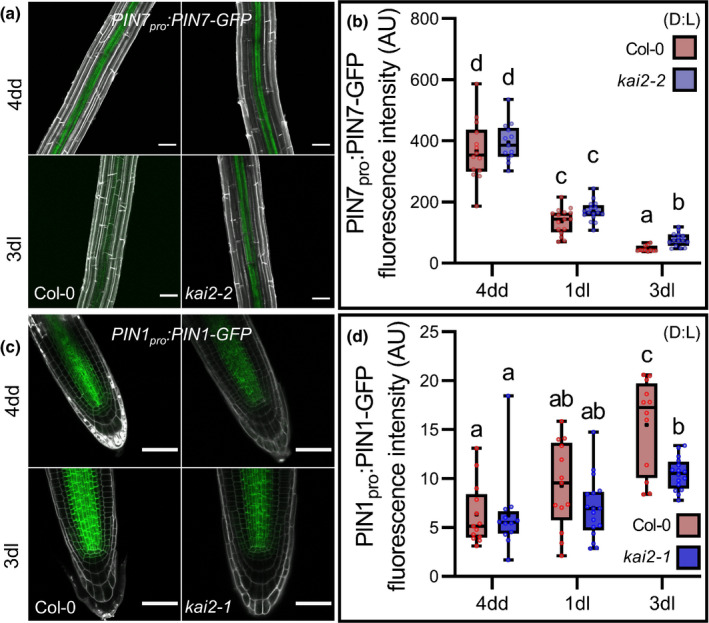
KAI2 mediates remodelling of auxin transport at the dark–light transition. For all figure panels (D) indicates *Arabidopsis thaliana* plants were grown in continuous darkness, (L) indicates that plants were grown under a standard light regime (16 h : 8 h, light : dark), and (D : L) indicates that plants were grown in continuous darkness for some days before transfer to standard light conditions. (a, b) PIN7‐GFP abundance quantification (b) and representative images (a) in the old differentiation zone (ODZ) of wild‐type or *kai2* roots after 4 d growth in the dark (4dd), and subsequent transfer to normal light conditions for 1 and 3 d (1dl, 3dl). Data correspond to two independent experimental replicates pooled together (*n* = 11–16 seedlings per genotype and time point). Statistical groups indicated by letters were determined by one‐way ANOVA with *post hoc* Tukey HSD (95% confidence interval (CI)). (b) Microscopy images overlay propidium iodide (grey) and GFP signals (green). Bar, 50 µm. (c, d) PIN1 : GFP abundance quantification (d) and representative images (c) in root meristem zone of wild‐type or *kai2* seedlings after 4 d growth in the dark (4dd), and subsequent transfer to normal light conditions for 1 and 3 d (1dl, 3dl). Data correspond to two independent experimental replicates pooled together (*n* = 12–17 seedlings per genotype and time point. Statistical groups indicated by letters were determined by one‐way ANOVA with *post hoc* Tukey’s honest significant difference (HSD) (95% CI). (d) Microscopy images overlay propidium iodide (grey) and green fluorescent protein (GFP) signals (green). Bar, 50 µm. (b, d) The boxes in the box plot show the lower and upper quartiles and median values, mean is represented as (•), whiskers show minimal and maximal data values.

We next tested whether this re‐organisation was delayed in *kai2* mutants, consistent with the changes in auxin distribution that we had observed (Fig. 2). In hypocotyls we observed a delay in the reduction of PIN3 abundance in some experiments, but the imaging of hypocotyls was difficult (as they were too thick to reliably image the midplane section where PIN3, PIN4 and PIN7 are expressed) so we therefore focused on PIN abundance in the root. We observed no difference in PIN7 abundance in mature root tissues between wild‐type and *kai2‐2* at 4dd, but there was a clear failure to decrease PIN7 abundance in *kai2‐2* after transfer to the light, relative to wild‐type (Fig. 5a,b). Conversely, for PIN1 abundance in the MZ, we observed the opposite; there was no difference between wild‐type and *kai2‐1* at 4dd, but there was delay in the increase of PIN1 abundance at 4dd/1dl and 4dd/3dl (Fig. 5c,d). These differences are long‐lasting, and even young light‐grown *kai2* seedlings show increased PIN7 abundance along the root axis, decreased PIN1 in the RAM, and reduced PIN2 abundance in the elongation zone relative to wild‐type seedlings (Fig. S4a–e). Therefore, *kai2* mutants showed a general reduction in the rate at which the auxin transport system was remodelled after transition to the light.

We examined PIN gene expression in *kai2* mutants after transfer to the light, but we did not observe any major differences (i.e. > 1.5‐fold change) in PIN gene expression in *kai2* relative to Col‐0 at 4dd, 4dd/1dl or 4dd/3dl, although there were some minor, statistically significant differences in *PIN7* expression between Col‐0 and *kai2* (Fig. 4). We cannot rule out subtle spatial changes in PIN gene expression between Col‐0 and *kai2*, which were missed by performing qPCR on whole‐seedling RNA, and which accounted for the differences in PIN protein abundance. However, given the similarity in expression pattern between Col‐0 and *kai2* we feel that the most parsimonious explanation for the data are that *kai2* mutants undergo similar light‐mediated changes in PIN transcription as Col‐0, but are sluggish in responding to these transcriptional changes.

Consistent with this delay in remodelling auxin transport after exposure to light, we also observed a delay in changes to *DR5v2:GFP* expression in *kai2* seedlings. Whereas wild‐type seedlings grown for 4 d in the dark showed a strong reduction of *DR5v2* expression in hypocotyls after 1 d of light exposure, and a gradual increase in *DR5v2* expression in the RAM over 3 d of light exposure, these changes did not occur in *kai2* mutants (Fig. S3). Indeed, if anything, *DR5v2* expression declined in the RAM after transfer to the light. Therefore, the observed reduction in auxin transport remodelling in *kai2* mutants after transfer to light delayed the changes in auxin response in seedling tissues, and we hypothesised this led to the observed phenotypes in *kai2* mutants.

### The phenotypic effects of KAI2 signalling are mediated by PIN‐mediated auxin transport

**Fig. 6:**
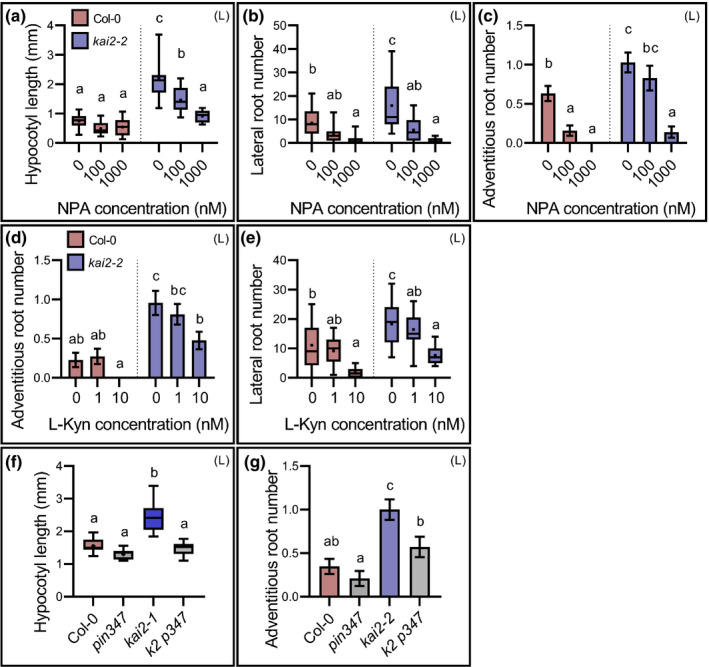
The phenotypic effects of KAI2 signalling are mediated by PIN‐mediated auxin transport. For all figure panels (D) indicates *Arabidopsis thaliana* plants were grown in continuous darkness, (L) indicates that plants were grown under a standard light regime (16 h : 8 h, light : dark), and (D : L) indicates that plants were grown in continuous darkness for some days before transfer to standard light conditions. (a–c) Effect of auxin transport inhibitor 1*‐N‐*naphthylphthalamic acid (NPA) on hypocotyl length (a) lateral root number (b) and adventitious root number (c) in 10‐d‐old wild‐type and *kai2‐2* seedlings. For (a) data correspond to one experimental replicate (*n* = 11–13 seedlings per genotype and treatment); two other independent experimental replicates gave comparable results; for (b) data correspond to one experimental replicate (*n* = 18–22 seedlings per genotype and treatment); a second independent experimental replicate gave comparable results; for (c) data correspond to one experimental replicate (*n* = 38–42 seedlings per genotype and treatment); a second independent experimental replicate gave comparable results. Statistical groups indicated by letters were determined by one‐way ANOVA with *post hoc* Tukey’s honest significant difference (HSD) (95% confidence interval (CI)). (c) Error bars represent ± SE. (d, e) Effect of auxin biosynthesis inhibitor l‐kynurenine (l‐KYN) on adventitious (d) and lateral root (e) number in 10‐d‐old wild‐type and *kai2‐2* seedlings. Data correspond to one experimental replicate (*n* = 21–22 seedlings per genotype and treatment); a second independent experimental replicate gave comparable results. Statistical groups indicated by letters were determined by one‐way ANOVA with *post hoc* Tukey HSD (CI 95%). (d) Error bars represent ± SE. (f, g) Hypocotyl length (f) and adventitious root number (g) in 10‐d‐old wild‐type, *kai2‐2*, *pin3‐3 pin4‐3 pin7‐1 (pin347) and kai2‐2 pin3‐3 pin4‐3 pin7‐1 (k2 p347)* seedlings. For (f) data correspond to one experimental replicate (*n* = 11–14 seedlings per genotype); a second independent experimental replicate gave comparable results; for (g) data correspond to two independent experimental replicates pooled together (*n* = 40–45 seedlings per genotype). Statistical groups indicated by letters were determined by one‐way ANOVA with *post hoc* Tukey HSD (95%CI). Error bars represent ± SE. (a, b, e, f) The boxes in the box plot show the lower and upper quartiles and median values, mean is represented as (•), whiskers show minimal and maximal data values.

Our data supported the idea that the altered auxin distribution we observed in *kai2* (Fig. 2) was caused by a failure to remodel the auxin transport system after dark–light transition (Fig. 3), and strongly suggested that this caused the accompanying failure to remodel seedling development after dark–light transition. To test this model, we used the auxin transport inhibitor 1*‐N‐*naphthylphthalamic acid (NPA) (Abas *et al*., 2021) to try and rescue the *kai2* phenotype. Consistent with our model, treatments in the range 0.1–1 µM NPA were sufficient to reduce the light‐grown hypocotyl, adventitious root, and lateral root phenotypes of *kai2* to a wild‐type level (Fig. 6a–c). We were able to achieve the same effect using the auxin synthesis inhibitor l‐kynurenine (He *et al*., 2011) at a concentration of 10 nM (Fig. 6d,e). To provide independent verification of these results, we crossed *kai2‐2* to the *pin3‐3 pin4‐3 pin7‐1* triple mutant (Bennett *et al*., 2016b; van Rongen *et al*., 2019). The verified quadruple mutant (*k2 pin347*) restored the light‐grown hypocotyl phenotype to a wild‐type level (Fig. 6f). The adventitious root phenotype of *kai2* was also rescued in the quadruple mutant (Fig. 6g), but the lateral root phenotype was harder to assess as *pin3 pin4 pin7* drastically reduced lateral root formation in a wild‐type background.

## Discussion

### A model for the function of KAI2 in seedling development

**Fig. 7:**
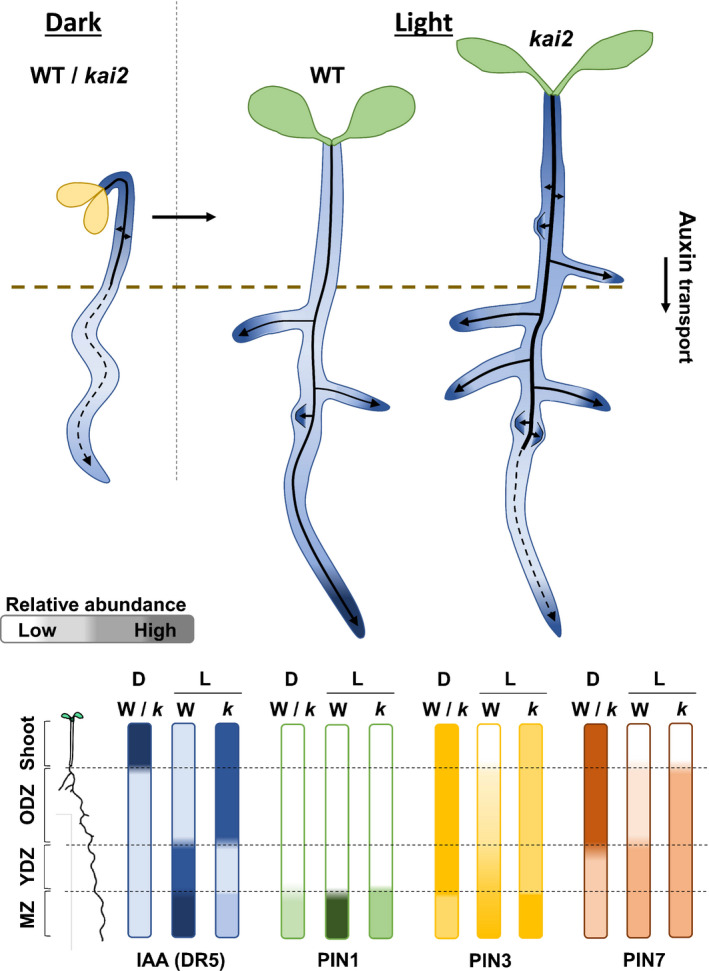
A model for KAI2 function in photomorphogenesis. Proposed model for light‐induced remodelling of the auxin transport system to regulate seedling development in *Arabidopsis thaliana*. During skotomorphogenesis, seedlings have a strong rootward auxin transport from the shoot apex, mediated by high PIN3 and PIN7 abundance, which drives the elongation growth of the hypocotyl and primary root. At the transition to photomorphogenic development, KAI2 mediates the rapid remodelling of the PIN‐mediated auxin transport system, with a reduction of PIN3 and PIN7 abundance in older tissues, and increased PIN1, PIN3 and PIN7 abundance in the root meristem, to promote a meristematically mediated growth in the light. In *kai2* mutants, the failure to remodel the auxin transport system transition leads to excess auxin in the shoot and older root tissues promoting continued hypocotyl elongation and increased adventitious and lateral roots growth in the shootward part of the root, with reduced auxin delivery to the primary root meristem. Solid arrows represent main auxin transport stream, dashed arrows represent reduced auxin transport; for the purpose of the model, the seedlings root tissues are compartmented as old differentiation zone (ODZ), young differentiation zone (YDZ), root meristem zone (MZ).

Taken together, these data are consistent with a model in which KAI2 mediates light‐induced remodelling of the auxin transport system to regulate seedling development. In the dark, there is a strong auxin transport connection between the shoot apex and cotyledons and the root, with high PIN3, PIN4 and PIN7 abundance along the main shoot–root axis of the plant, and low PIN1/PIN2 abundance in the root meristem (Fig. 7). Intriguingly, this auxin transport system does not seem to be particularly important for skotomorphogenic development (consistent with Jensen *et al*., 1998), as *pin3 pin4 pin7* etiolates normally. Its function might be related more to the delivery of auxin to the root system for future growth, than to growth in the dark (Fig. 7). After the transition to light, this system is rapidly remodelled, with the turnover of PIN3, PIN4 and PIN7 in the hypocotyl and older root tissues, and upregulation of PIN1, PIN2, PIN3 in the root meristem (Fig. 7). PIN1 upregulation may act to ‘inject’ auxin into the meristem from the rest of the root, driving cell division, consistent with the strong increase in meristem size that occurs in the light (Fig. 1g) (Sassi *et al*., 2012). In addition, PIN2, PIN3, PIN4 and PIN7 upregulation, in concert with AUX1 (Villaécija‐Aguilar *et al*., 2021a), promotes the ‘reflux’ of auxin from the root cap to the epidermis, which drives elongation zone activity and RH development (Fig. 7). Collectively, this remodelling ‘kick‐starts’ the meristematic ‘engine’ of growth, allowing a move away from primarily elongation‐driven growth in the dark. However, in *kai2* mutants, the failure to quickly remodel the system leads to continued auxin transport from cotyledons into the hypocotyl, delaying the expansion of the cotyledons, and promoting continued hypocotyl elongation, and the formation of adventitious roots (Fig. 7). This excess auxin reaches the upper and older parts of the root system, where it promotes increased lateral root initiation and emergence. Interestingly, we found that, while there is *increased* transport from the shoot to the upper root, there is then *reduced* transport of auxin from the upper root to the root meristem in *kai2* roots, relative to Col‐0 (Fig. S5a), presumably because it is diverted into the LR, which show increased auxin reporter activity relative to wild‐type (Fig. S2a,b). Consistent with this reduced overall transport, we found that auxin reporter activity was diminished in the primary root meristem of *kai2‐2*, the opposite to that seen in the LR (Fig. S5b,c). This reduced auxin delivery to the primary root meristem is likely to account for some of the phenotypes observed in the primary root in *kai2* (Villaécija‐Aguilar *et al*., 2019).

### New perspectives on KAI2 signalling

The data presented here provide a holistic explanation for the role of KAI2 in seedling growth. It has long been speculated that KAI2 targets the SMAX1 (SUPPRESSOR OF MAX2 LIKE 1) and SMXL2 proteins for degradation, analogous to the D14–SMXL7/D53 interaction (Machin *et al*., 2020), and recent data provide confirmation of this idea (Khosla *et al*., 2020; Wang *et al*., 2020b). However, apart from an increase in ethylene biosynthesis in the root (Sami *et al*., 2019; Carbonnel *et al*., 2020), KAI2‐mediated signalling events downstream of SMAX1/SMXL2 remain unclear. There are some well established genes upregulated in response to KAI2 signalling, including its homologue *DWARF14‐LIKE2* (*DLK2*) and *KAI2‐UPREGULATED F‐BOX1* (*KUF1*) (Waters *et al*., 2012), but the function of these genes remains enigmatic, and DLK2 has no obvious role in development (Vegh *et al*., 2017). One possibility is that KAI2 might regulate the efficient allocation of PIN auxin transporters to the plasma membrane, analogous to the role of D14 in the shoot (Crawford *et al*., 2010; Shinohara *et al*., 2013), although more work would be needed to demonstrate this. This could be consistent with recent data showing that MAX2 and *rac*‐GR24 signalling inhibits the inhibitory effect of auxin on PIN endocytosis in roots (Zhang *et al*., 2020). Although Zhang and colleagues suggested that these effects reflected the output of strigolactone signalling, they might equally reflect outputs of KAI2 signalling, given the use of *max2* mutants and *rac*‐GR24 in these experiments, which does not allow the distinction between KAI2 and D14‐mediated signalling (Waters *et al*., 2012; Machin *et al*., 2020).

By promoting this efficient cellular remodelling of auxin transport, KAI2 promotes a larger‐scale remodelling of the auxin transport system, allowing seedlings to undergo rapid changes in growth in response to light exposure. In the shoot, the mechanism by which D14‐mediated signalling events in the nucleus leads to changes in PIN protein allocation/removal from membranes is unclear (Liang *et al*., 2016). The effect of D14 on PIN proteins does not involve changes in PIN gene expression, and is unaffected by cycloheximide (Shinohara *et al*., 2013). Our data are consistent with the effect of KAI2 on PIN proteins being mediated independently of PIN transcription, but it is nevertheless possible that KAI2 might act transcriptionally on other genes, which in turn regulate PIN allocation to the membrane. There is certainly increasing evidence for the role of SMXL7/D53 proteins as transcriptional co‐repressors (Wang *et al*., 2020a), but not all their effects can be explained by a simple transcriptional co‐repression model (Liang *et al*., 2016). However, the ability to use the seedling‐based system described here will greatly simplify future investigations of downstream SMXL function, both transcriptional and nontranscriptional.

## Author contributions

TB, OL and CG designed the study. MH‐J, JAVA, KL and TB planned and carried out experiments and analysed data. MH‐J and TB wrote the manuscript with input from all authors.

## Supporting information


**Fig. S1** KAI2 mediates light‐induced remodelling of seedling development.
**Fig. S2** KAI2 modulates auxin distribution in the seedling.
**Fig. S3** Remodelling of auxin distribution/response at the dark–light transition.
**Fig. S4** KAI2 mediates remodelling of auxin transport at the dark–light transition.
**Fig. S5** A model for KAI2 function in photomorphogenesis.
Please note: Wiley Blackwell are not responsible for the content or functionality of any Supporting Information supplied by the authors. Any queries (other than missing material) should be directed to the *New Phytologist* Central Office.

## Data Availability

All data associated with this manuscript will be made available upon request.
