# Supplementary material for: KAI2 regulates seedling development by mediating light‐induced remodelling of auxin transport
Source: New Phytol. 2022 Apr 9;235(1):126–40. doi: 10.1111/nph.18110 (PMC9320994; doi:10.1111/nph.18110)
Supplement: Supplementary file 1 — Fig. S1 KAI2 mediates light‐induced remodelling of seedling development. Fig. S2 KAI2 modulates auxin distribution in the seedling. Fig. S3 Remodelling of auxin distribution/response at the dark–light transition. Fig. S4 KAI2 mediates remodelling of auxin transport at the dark–light transition. Fig. S5 A model for KAI2 function in photomorphogenesis. Please note: Wiley Blackwell are not responsible for the content or functionality of any Supporting Information supplied by the authors. Any queries (other than missing material) should be directed to the New Phytologist Central Office. [file NPH-235-126-s001.pdf]

New Phytologist **Supporting Information**

Article title: **KAI2 regulates seedling development by mediating light-induced remodelling of auxin transport**

Authors: Maxime Hamon-Josse, Jose Antonio Villaecija Aguilar, Karin Ljung, Ottoline Leyser, Caroline Gutjahr, Tom Bennett

Article acceptance date: 05 March 2022

The following Supporting Information is available for this article:

Fig. S1 KAI2 mediates light induced remodelling of seedling development

Fig. S2 KAI2 modulates auxin distribution in the seedling

Fig. S3 Re-modelling of auxin distribution/response at the dark-light transition

Fig. S4 KAI2 mediates re-modelling of auxin transport at the dark-light transition

Fig. S5 A model for KAI2 function in photomorphogenesis

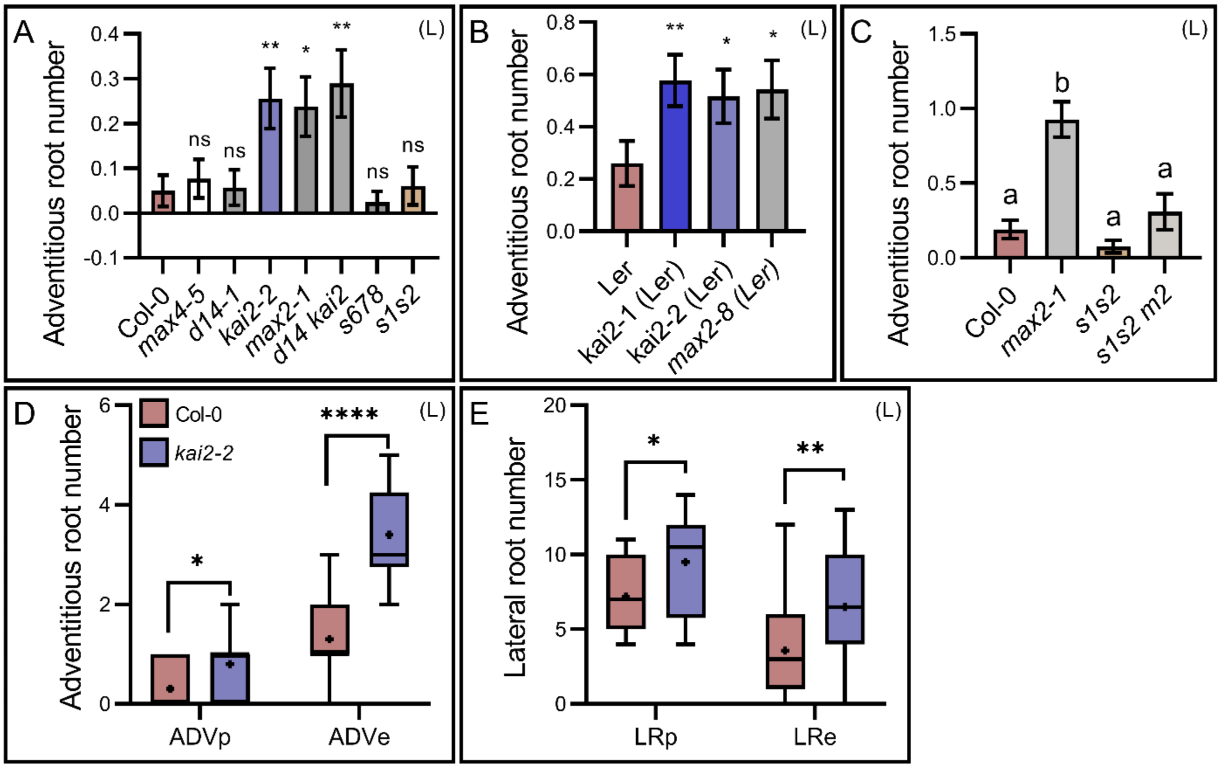

Fig. S1 For all figure panels (L) indicates that Arabidopsis plants were grown under a standard light regime (16 hours light, 8 hours dark). (**A**) Adventitious and junction root number in 10-day old light-grown seedlings impaired in SL biosynthesis, signalling, downstream targets (respectively, *max4-5*, *d14-1*, *smxl6 smxl7 smxl8* (*smxl678*)), KL signalling and downstream target (*kai2-2*, *smax1 smxl2 (s1s2), smax2 smxl2 max2*) or SL and KL signalling (*d14 kai2*, *max2-1*). Data correspond to one experimental replicate (n=43-91 seedlings per genotype), three other independent experimental replicates gave comparable results. *,** (p-value ≤ 0.05, 0.01) indicates differences compared to wild-type (Welch's t-test). ns = no significant difference. Error bars represent ± s.e.m. (**B-C**) Adventitious and junction root number in 10-day old light-grown seedlings of KL signalling mutants in Lansberg erecta (Ler) ecotype (**B**), and KL signalling mutants in Columbia ecotype (**C**). Data correspond to two independent experimental replicates pooled together (n=19-44 seedlings per genotype). In (**B**), *,** (p-value ≤ 0.05, 0.01) indicates differences compared to wild-type (Welch's t-test). In (**C**), letters represent statistical groups determined by one-way ANOVA with post hoc Tukey HSD (CI 95%). Error bars represent ± s.e.m. (**D**) Number of primordia and emerged adventitious roots in 11-day old de-rooted and etiolated Col-0 or *kai2-2* seedlings. Data correspond to one experimental replicate (n=10 seedlings per genotype); two other independent experimental replicates gave comparable results. *,**** (p-value ≤ 0.05, 0.0001) indicates differences compared to wild-type (Welch's t-

test). (**E**) Number of lateral root primordia and emerged lateral roots in 10-day old light-grown seedlings. Data correspond to three independent experimental replicates pooled together (n=11-30 seedlings per genotype). *,** (p-value ≤ 0.05, 0.01) indicates differences compared to wild-type (Welch's t-test). (**D-E**) The boxes in the box plot show the lower and upper quartiles and median values, mean is represented as (●), whiskers show minimal and maximal data values.

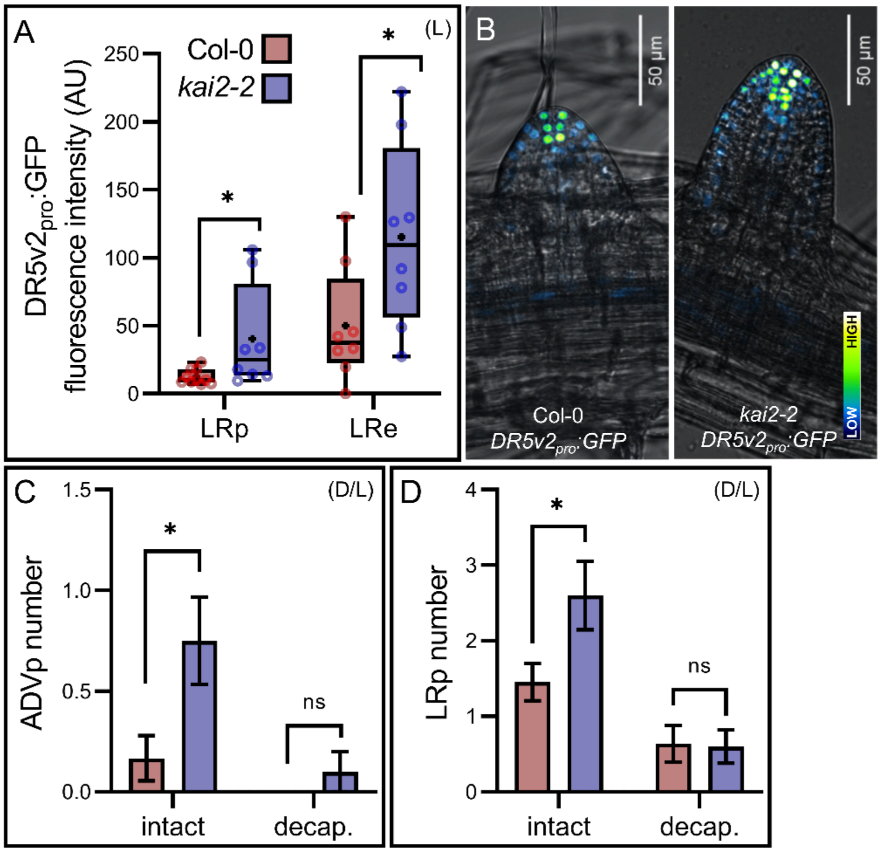

Fig. S2 For all figure panels (L) indicates that Arabidopsis plants were grown under a standard light regime (16 hours light, 8 hours dark), and (D/L) indicates that plants were grown in continuous darkness for a number of days before transfer to standard light conditions.

(**A**-**B**) Auxin response quantification (averaged *DR5v2:GFP* fluorescence intensity for 5 nuclei per tissue) in lateral roots of Col-0 and *kai2* seedlings. (**A**) and representative images (**B**) in lateral roots of WT and *kai2-2* seedlings grown for 6 days under normal light conditions. Data correspond to the average GFP intensity in 8-10 lateral root primordia of similar stage, and 8-10 emerged lateral roots of similar stage, taken from 5 seedlings per genotype, where each sample is the average of 5 nuclei, from one experimental replicate;, two other independent experimental replicates gave comparable results. * (p-value ≤ 0.05) indicates differences compared to wild-type (Welch's t-test). (**A**) The boxes in the box plot show the lower and upper quartiles and median values, mean is represented as (●), whiskers show minimal and maximal data values. (**B**) Microscopy images overlay bright field (grey) and GFP-derived signals. GFP signal is represented with false-colour with dark blue as low signal intensity and bright white as high signal intensity. Scale bars represent 50 µm.

(**C**-**D**) Adventitious (**C**) and lateral (**D**) root primordia number per seedling grown in the dark for 4 days and subsequently undergoing apex de-capitation (decap.) or left intact (intact) before transfer to normal light conditions for 3 days. Data correspond to one experimental replicate (n = 10-12 seedlings per genotype and treatment); a second independent experimental replicate gave comparable results. * (p-value ≤ 0.05) indicates differences compared to wild-type (Welch's t-test). ns = no significant difference. Error bars represent ± s.e.m.

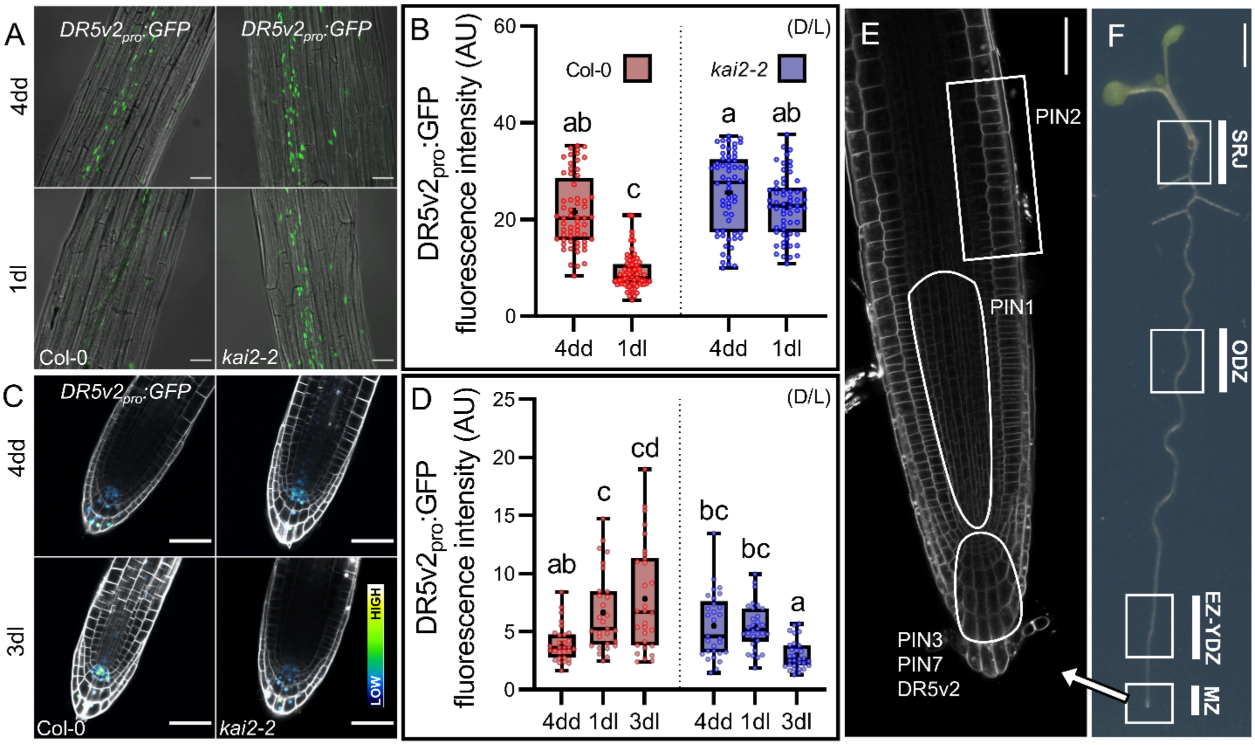

Fig. S3 Re-modelling of auxin distribution/response at the dark-light transition For all figure panels (D/L) indicates that Arabidopsis plants were grown in continuous darkness for a number of days before transfer to standard light conditions. (**A**-**D**) Auxin response (*DR5v2:GFP* fluorescence intensity) in the hypocotyl (**A**, **B**) and root apical meristem (**C**, **D**) of seedlings of wild-type or *kai2-2* mutants grown 4 days in the dark and then transferred in light condition for 1 day (top row) or 3 days (bottom row) (1dl, 3dl). (**A**) and (**C**) show representative microscopy images with overlay of either bright field or propidium iodide staining (grey) and GFP signals represented in green (**A**) or with false colour with dark blue as low signal intensity and bright white as high signal intensity (**C**). Scale bars represent 50 µm. (**B**) GFP quantification in the hypocotyl; Data correspond to one experimental replicate (n=60-70 nuclei measured out of 6-7 seedlings of each genotype); a second independent experimental replicate gave comparable results. (**D**) GFP quantification in the RAM; Data correspond to one experimental replicate (n=29-30 nuclei measured out of 3-5 seedlings of each genotype. (**B**,**D**) Statistical groups indicated by letters were determined by one-way ANOVA with post hoc Tukey HSD (CI 95%). The boxes in the box plot show the lower and upper quartiles and median values, mean is represented as (●), whiskers show minimal and maximal data values. (**E**) Root tip regions used for measurements in Figure 3, 4, 5, and

supplemental figure 3, 4, 5. PIN2-GFP signal is measured in the epidermis from 210 µm to 250 µm from the root tip. PIN1-GFP signal is measured in the stele from 70 µm to 150 µm from the root tip. PIN3-GFP, PIN7-GFP, and (DR5v2:)GFP signals are measured in the apical root meristem. Scale bar represents 50 µm. (**F**) Seedling tissues, shoot-root junction (SRJ), older differentiation zone (ODZ), junction of the elongation and young differentiation zone (EZ-YDZ), and meristem zone (MZ), used for PIN3-GFP and PIN7-GFP signal measurements in figure 3 and supplemental figure 4. Scale bar represents 10 mm.

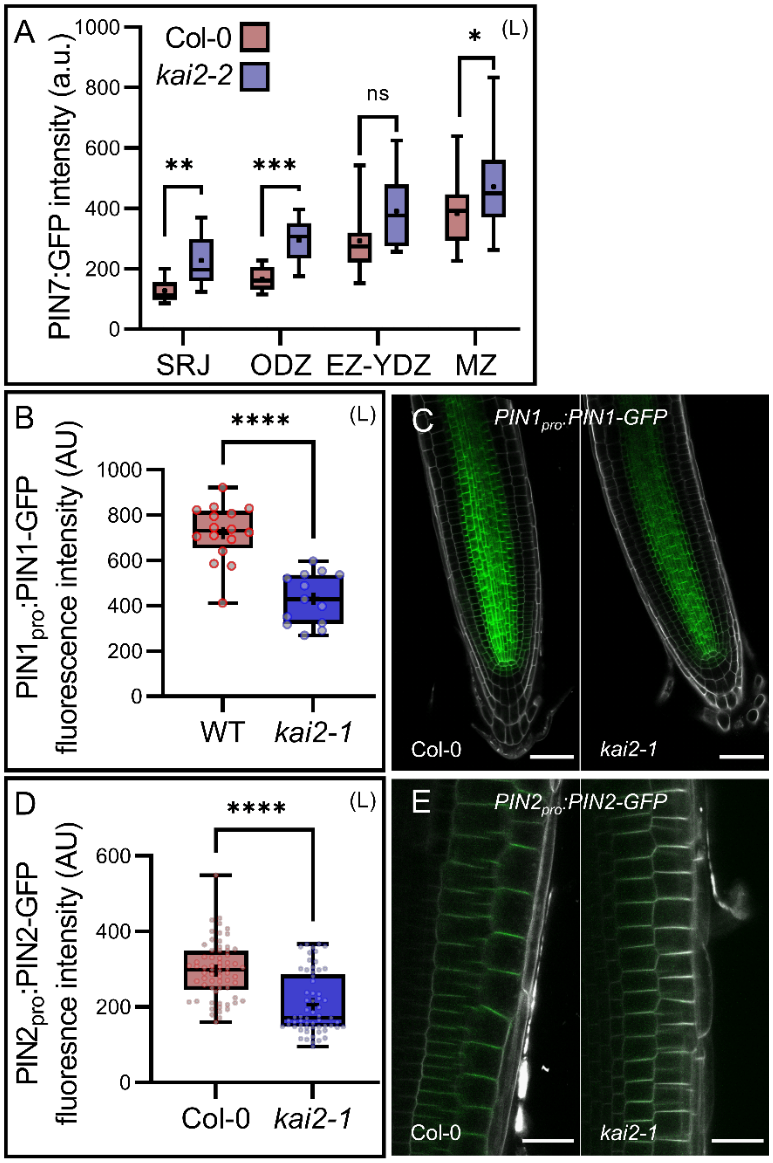

Fig. S4 KAI2 mediates re-modelling of auxin transport at the dark-light transition. For all figure panels (L) indicates that Arabidopsis plants were grown under a standard light regime (16 hours light, 8 hours dark). (**A**) Quantification of PIN7-GFP signal in the shoot-root junction (SRJ), older differentiation zone (ODZ), junction of the elongation and young differentiation zone (EZ-YDZ), and meristem zone (MZ) of 6-day old wild-type and *kai2-2* seedlings grown under normal light conditions. Data correspond to one experimental replicate (n=8-18 seedlings per genotype); a second independent experimental replicate gave comparable results. *,**,*** (p-value ≤ 0.05, 0.01, 0.001) indicates differences compared to wild-type (Welch's t-test). ns = no significant difference. (**B**,**C**) PIN1-GFP quantification (**B**) and representative microscopy

images (**C**) in the meristem zone of 6-day old wild-type and *kai2-1* seedlings grown under normal light conditions. Data correspond to one experimental replicate (n=13-16 seedlings per genotype); two other independent experimental replicates gave comparable results. **** (p-value ≤ 0.0001) indicates differences compared to wild-type (Welch's t-test). (**C**) Microscopy images overlay propidium iodide staining (grey) and GFP-derived signal represented in green. Scale bars represent 50 µm. (**D**, **E**) PIN2-GFP quantification (**D**) and representative microscopy images (**E**) in the apical plasma membrane of epidermal cells in the meristematic zone of 4-day old wild-type and *kai2-1* seedlings grown under normal light conditions. Data correspond to one experimental replicate (n = 61-65 plasma membranes from 5-6 seedlings of each genotype. **** (p-value ≤ 0.05, 0.01, 0.001, 0.0001) indicates differences compared to wild-type (Welch's t-test). (**E**) Microscopy images overlay propidium iodide staining (grey) and GFP-derived signal represented in green. Scale bars represent 20 µm. (**A,B,D**) The boxes in the box plot show the lower and upper quartiles and median values, mean is represented as (●), whiskers show minimal and maximal data values.

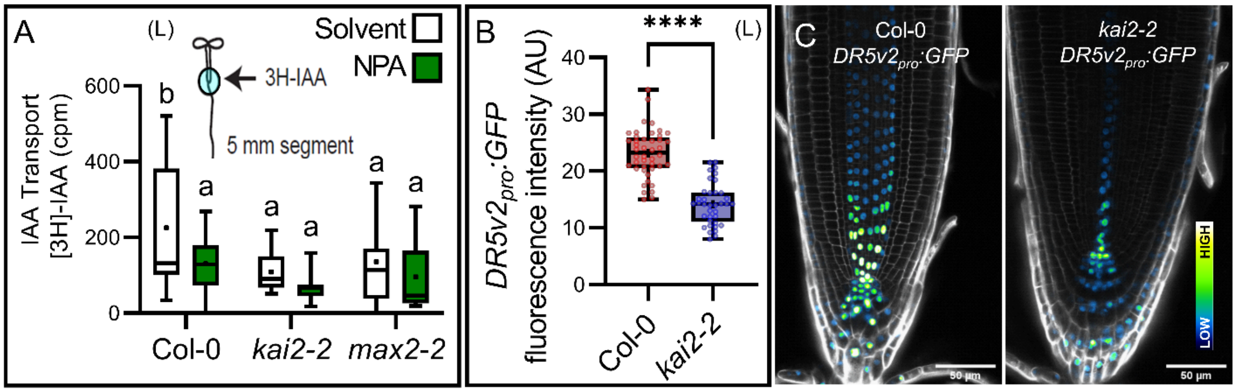

Fig. S5 A model for KAI2 function in photomorphogenesis. For all figure panels (L) indicates that Arabidopsis plants were grown under a standard light regime (16 hours light, 8 hours dark). (**A**) Basipetal ^{3}H-IAA auxin transport in 5-day old seedlings. Arrow indicates the site of ^{3}H-IAA or solvent application at the shoot-root junction, radioactivity was measured in a 5 mm segment at the primary root meristem. Data correspond to one experimental replicate (n = 10-13 seedlings per genotype and treatment), a second independent experimental replicate gave comparable results. Statistical groups indicated by letters were determined by one-way ANOVA with post hoc Tukey HSD (CI 95%). (**B**,**C**) Auxin response (*DR5v2:GFP* fluorescence intensity) in root meristem zone of seedlings in normal light-grown conditions. (**B**) Shows GFP signal quantification and (**C**) representative images. Data correspond to three independent experimental replicates pooled together (n = 37-44 seedlings per genotype). **** (p-value ≤ 0.0001) indicates differences compared to wild-type (Welch's t-test). (**C**) Microscopy images overlay propidium iodide staining (grey) and GFP-derived signal represented with false colour with dark blue as low signal intensity and bright white as high signal intensity. Scale bars represent 50 µm. (**A-B**) The boxes in the box plot show the lower and upper quartiles and median values, mean is represented as (●), whiskers show minimal and maximal data values.
